# Mass distribution of azithromycin to prevent under-five mortality in sub-Saharan Africa: Do the benefits outweigh the costs with regard to antimicrobial resistance?

**DOI:** 10.34172/hpp.2022.15

**Published:** 2022-08-20

**Authors:** Sodiq Inaolaji Yusuff, Iyiola Olatunji Oladunjoye, Olalekan Tolulope Popoola, Bolarinwa Olufunmilayo Victoria

**Affiliations:** ^1^Department of Medicine, Faculty of Clinical Science, Obafemi Awolowo University, Ile-Ife, Nigeria; ^2^Department of Microbiology, Faculty of Life Sciences, University of Ilorin, Ilorin, Nigeria; ^3^Department of Pharmacy, Faculty of Pharmacy, Madonna University, Nigeria

## Dear Editor,

 Sub-Saharan Africa (sSA) accounts for the highest burden of under-five mortality (U5M) globally, with an estimated under-five mortality rate (U5MR) of 76 deaths per 1000 live births in 2018, leading to approximately 2.7 million deaths.^1^ The region alone accounts for over 55% of the global under-five deaths despite having less than 30% of the world’s under-five population.^2^ Sharrow et al^2^ estimated that about 75% of the 53 countries that are not likely to attain the SGD-3 U5MR target are in sSA, necessitating the need to intensify efforts in these countries to meet the SDG-3 target. As part of the intensified efforts, different interventions targeted at sSA’s under-five population are being considered or have been introduced in recent years. One of those is the mass distribution of azithromycin (MDA-azithromycin) to under-five children, which has shown to reduce U5M in sSA. It is postulated that since infectious disease is a leading cause of U5M in sSA, with malaria, diarrhoea, and pneumonia jointly accounting for about 40% of U5M in the region and azithromycin being effective against these diseases,^1,3^ perhaps MDA-azithromycin could be a cost-effective public health intervention to mitigate the overwhelming U5MR burden in sSA. Community-randomized trial in Ethiopia and MORDOR clinical trials across 3 sSA countries, namely Malawi, Tanzania, and Niger, on MDA-azithromycin showed reduction in U5MR by 50% and 14% respectively compared to placebo.^1^ As a result, in 2018, the WHO developed a guideline to guide the population-level implementation of MDA-azithromycin in countries with high U5M and a heavy burden of morbidities due to diarrhoea, pneumonia and malaria. Some sub-Saharan African countries have put plans in motion to roll-out the intervention. However, a pertinent question that keeps lingering is the potential negative attendant effects of MDA-azithromycin on anti-microbial resistance.

 The new Global Research on Antimicrobial Resistance (GRAM) reported that over 4.95 million deaths were attributable to AMR in 2019, with the highest case seen in western sSA at 27 deaths per 100 000 compared to Australasia, which had the least at 6.5 deaths per 100 000.^4^ The review on antimicrobial resistance (AMR) predicted that AMR could kill 10 million people per year by 2050, and sSA would contribute significantly to this number. With this, it is essential to critically analyze all possible interventions and practices that could contribute to the AMR burden in this region. Hence, a relevant question worth asking is “Will the population-level implementation of MDA-azithromycin exacerbate AMR-related issues in sSA?” While there is no clear-cut answer yet, evidence has shown that there is a possibility of a perpetual increase in macrolide and other antibiotic resistance in gut and respiratory bacteria after repeated MDA-azithromycin.

 Following MDA-azithromycin for trachoma control in rural Tanzania, Seidman et al demonstrated a significant increase in carriage of macrolide-resistant *E. coli *strains by 400% in young children.^5^ Similarly, 24 months after the commencement of the MORDOR clinical trial, Doan et al showed higher proportion of macrolide-resistant nasopharyngeal *S. pneumonia *in azithromycin-treated communities (mean 12.3%, 95% confidence interval 5.7—20.0%) than in the placebo-treated communities (2.9%, 0—6.1%, *P* = 0.02).^6^ In the same vein, azithromycin-treated communities also had more macrolide-resistant determinants in the guts (68.1%, 60.4—74.8% vs 46.3%, 35.9—52.9%; *P* < 0.001). Other studies also demonstrated marked rises in azithromycin-resistant pneumococcal isolates with a baseline macrolide-resistant rate of < 5% rising to 80% subsequent to multiple mass azithromycin treatments.^1^ Although the resistance rates decreased after cessations of MDA-azithromycin across the different studies, they did not return to the baseline levels again. Further, there are inconsistent reports on the effects of MDA-azithromycin on other classes of antibiotics. For instance, Doan et al described a significant increase in pneumococcal clindamycin resistance after MDA, whereas Skalet et al^7^ showed a non-consequential pattern of increasing clindamycin resistance after multiple rounds of MDA-azithromycin. Similar inconsistent trends were seen in pneumococcal resistance to sulfamethoxazole or chloramphenicol after MDA azithromycin. Thus, there is a need to conduct more elaborate studies to fully understand the effects of MDA-azithromycin on AMR before scaling up the intervention across sSA.

 While MDA-azithromycin has been effective in addressing trachoma, contributing to the elimination of the disease in 14 countries as of March 2022, the impact of the intervention on U5M reduction is inconsistent. Although the MORDOR clinical trials, where children aged 1-59 months received bi-annual dosage of azithromycin over four years through mass campaigns in 3 sSA countries, showed an overall reduction in mortality by 14%, the outcomes varied across different age groups and countries. Majority of the under-five deaths averted were seen in Niger with about 18% reduction—the only site with a statistically significant U5M reduction—whereas Malawi and Tanzania had barely 5.7% and 3.5% reductions respectively.^8^ Also, while significant reduction in mortality was recorded in the infant age group, about 25%, the intervention was of little benefits in children aged 1-5 years. The overall 14% reduction in all-cause mortality is significantly lower than that seen in the community-randomized trial in Ethiopia, which recorded approximately 50% overall reduction. Another trial conducted in Mali and Burkina Faso, alongside seasonal malaria chemoprevention, showed no reduction in mortality or rate of hospitalization in children aged 3-59 months.^1^ Based on this evidence, the need for more research on MDA-azithromycin before scale-up is critical.

 Admittedly, MDA-azithromycin is a possible tool in accelerating SDG-3 attainment by preventing U5M in sSA, but certain measures need to be put in place before rolling out the interventions at a large scale to achieve the desired outcome. For one, it is quite critical to conduct more studies on the extent of MDA azithromycin’s effect on AMR. While many studies have confirmed macrolide resistance as an undesired effect of the intervention, data is still quite limited on the effects of MDA-azithromycin on other classes of antibiotics. Likewise, it is also crucial to increase the AMR surveillance capacity of the eligible countries to track and speedily detect emerging resistance. This is particularly important as most of the countries eligible for this intervention have weak disease surveillance systems. To further reduce the likelihood of AMR, it might be prudent to conduct more studies to ascertain whether the intervention should be targeted at infants or the whole under-five age group. Considering that MDA-azithromycin showed more impact in the infant aged group with insignificant effect on children aged 1-5 years, limiting the intervention to under-one children might be a major mitigating factor in reducing AMR risk. Another relevant tool would be utilizing mathematical modelling to forecast the effectiveness of introducing MDA-azithromycin on U5M reduction against the negative attendant effects of AMR on U5M and the general population. Conclusively, evidence suggests that MDA-azithromycin holds the potential of averting several thousands of child mortalities, a menace that has longed plagued Africa; however, policymakers must as a matter of necessity conduct policy-relevant studies to determine the extent of MDA azithromycin’s effect on AMR while judiciously establishing the guiding precepts and scope of the intervention to ensure it achieves the desired impact.

## Authors’ contributions

 SIY: Conceptualizing the topic; conducting literature review; and writing the manuscript. IOO: Conceptualizing the topic, reviewing the article, and finding the right journal. OTP: Drafting the recommendation and reviewing the manuscript. BOV: Conducting literature review, writing some sections of the article and revising the manuscript.

## Ethical approval

 This article does not require ethical approval as all the data gathered are from literature review.

## Competing interests

 There are no relevant financial or non-financial competing interests to report
